# The Direction of the Antibacterial Effect of Rutin Hydrate and Amikacin

**DOI:** 10.3390/antibiotics12091469

**Published:** 2023-09-21

**Authors:** Maria Miklasińska-Majdanik, Małgorzata Kępa, Tomasz J. Wąsik, Karolina Zapletal-Pudełko, Magdalena Klim, Robert D. Wojtyczka

**Affiliations:** 1 Department of Microbiology and Virology, Faculty of Pharmaceutical Sciences in Sosnowiec, Medical University of Silesia in Katowice, Jagiellońska 4, 41-200 Sosnowiec, Poland; mkepa@sum.edu.pl (M.K.); karolinazapletal@yahoo.com (K.Z.-P.); magdalena.klim@sum.edu.pl (M.K.); rwojtyczka@sum.edu.pl (R.D.W.); 2 Department of Medical Microbiology, Faculty of Medical Sciences in Katowice, Medical University of Silesia in Katowice, Medyków 18, 40-752 Katowice, Poland; twasik@sum.edu.pl

**Keywords:** rutin hydrate, amikacin, fractional inhibitory concentration

## Abstract

The aim of the presented study was to examine the in vitro antimicrobial activity of rutin hydrate (RH) alone and in combination with amikacin against 12 reference strains of Gram-positive and Gram-negative bacteria. The antibacterial activity assay was evaluated in the concentration range of 2–2048 µg/mL. A serial microdilution method was used to determine the minimal inhibitory concentration (MIC) of the examined compound against reference strains. RH showed varying potential against the tested strains with MICs ranging from 128 to 1024 µg/mL. In order to examine the combinatory profile of RH and amikacin, the fractional inhibitory concentrations (FICs) were determined. The RH–amikacin combination was more active against Gram-negative bacteria where four synergism and two additive interactions were noted. For four out of six Gram-positive isolates, an indifferent effect of RH and amikacin was demonstrated, and for two strains, the tested combination had an additive effect. The results of this study showed that RH possesses antimicrobial potential in vitro towards the tested reference isolates. Moreover, it shows a promising combined effect with amikacin against Gram-negative bacteria.

## 1. Introduction

Antimicrobial Resistance (AMR) is a significant global problem with serious public health risks. The treatment options and ability to control infections are gradually being exhausted, and the duration of infection and hospitalization has increased, all of which have led to an increase in mortality [1]. Due to the ever-growing problem of antibiotic resistance, scientists should focus their attention on finding alternatives to antibiotics as soon as possible. Searching for and testing new chemical compounds showing bacteriostatic or bactericidal activity will increase our chances of therapeutic success in infections caused by antibiotic-resistant bacteria [2,3].

**Figure 1:**
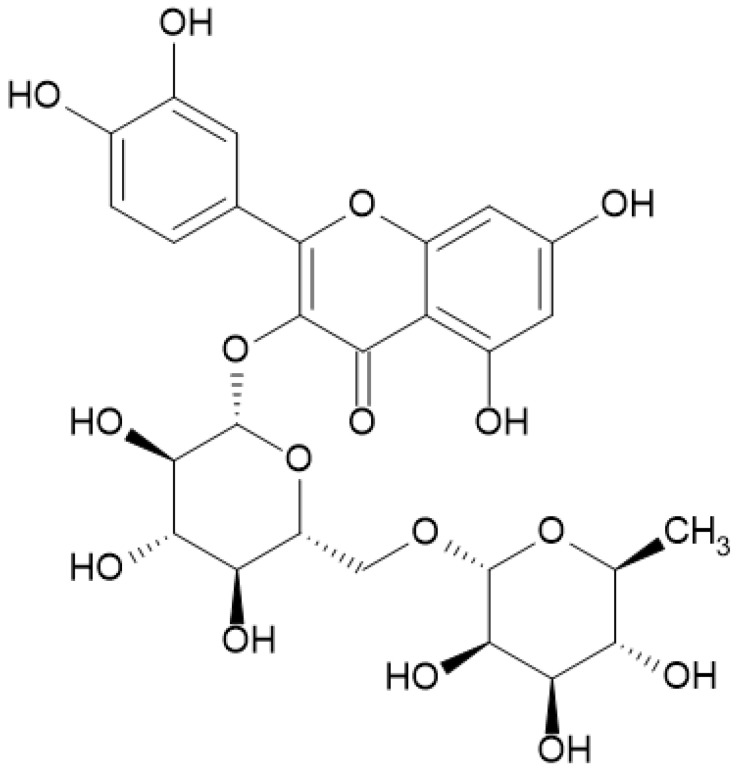
Chemical structure of rutin.

Many previous studies have shown that compounds of natural origin have antibacterial activity alone and in combination with selected antibiotics [4,5,6,7,8]. So far, in our research, we have focused on the antibacterial effect of phenolic acids and flavanols against staphylococcal strains. In this research, we studied the antimicrobial properties of rutin hydrate, which is a representative of glycoside flavonoids, against reference strains of Gram-positive and Gram-negative bacteria. Rutin is composed of aglycone and sugar residue (Figure 1). The aglycone part is composed of quercetin linked with an O-glycosidic bond with rutinose, which is a disaccharide [9,10]. Rutinoside was first isolated from the common rue herb (*Ruta graveolens* L.), from which the name of the organic chemical compound is derived. Some examples of the sources of rutin are Japanese pagoda tree flowers (*Styphnolobium japonicum*), buckwheat herb (*Fagopyrum esculentum*), and eucalyptus leaves (*Eucalyptus* spp.), among many others. Rutin is also commonly found in vegetables and fruits, mainly citrus fruits, and plant-based drinks [9]. Pure rutin takes the form of a yellow powder with a structure of fine crystals. Due to its lipophilic nature, rutoside is soluble in organic solvents, but very poorly soluble in water. To obtain a better assimilable form of rutin, various methods of raw material preparation are used, e.g., with hydration, hydrates are obtained, from which it is easier to prepare the desired solutions of rutin [10].

The structure of rutin determines its biological activity, which, in turn, influences its pharmacological properties. The main advantage of rutin is its antioxidant properties. Due to the presence of free hydroxyl groups, rutin has the ability to neutralize reactive oxygen species. By reducing free oxygen radicals, rutin protects the cells of the human body because it limits the formation of damage-causing mutations in the genetic material. In addition, quercetin-3-O-rutoside as an antioxidant prevents the peroxidation of phospholipids that form the cell membrane and consequently reduces the damage caused by free radicals. In this way, rutinoside directly contributes to the protection of cells against apoptosis caused by DNA damage or cell lysis as a result of cell membrane instability [11,12].

In addition, it has been proven that rutin exerts an antiatherogenic effect because it inhibits the oxidation of low-density lipoproteins and consequently reduces the formation of atherosclerotic plaques, which, in turn, have a protective effect on the cardiovascular system. Moreover, rutin slows down the oxidation of vitamin C, which means that ascorbic acid in the presence of rutin has a prolonged antioxidant effect. This property has been used in medical preparations and dietary supplements. Vitamin C, in addition to its important role as a free radical scavenger, is also a cofactor in proline and lysyl hydroxylases, which catalyze the hydroxylation of proline and lysine, respectively. Hydroxyproline and hydroxylysine are the main amino acids of collagen, which is an important structural element of blood vessels because it gives them durability and elasticity. In addition, rutin inhibits the activity of hyaluronidase, which is responsible for the digestion of collagen, and also reduces the activity of metalloproteinases responsible for the degradation of the extracellular matrix. All these properties contribute to the improvement of blood vessel conditions due to an increase in their elasticity and at the same time a reduction in their fragility and permeability [9].

The antibacterial properties of rutin have also been proven, which may find practical applications in the era of antibiotic resistance. Previous scientific research has demonstrated the antimicrobial activity of rutin against both Gram-positive and Gram-negative bacterial strains [13,14,15,16,17].

**Figure 2:**
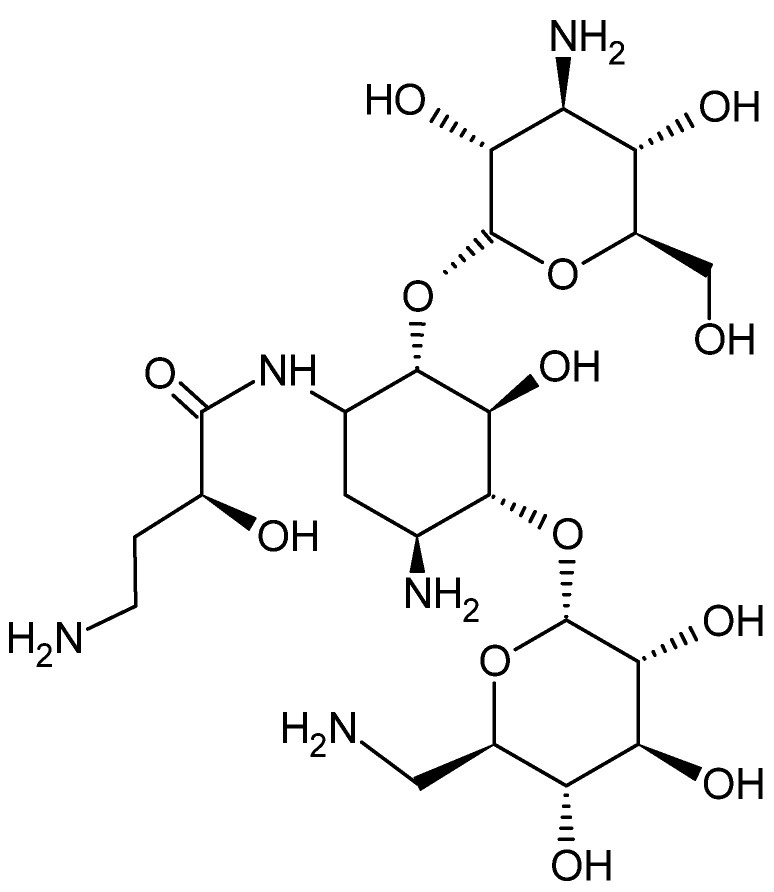
Chemical structure of amikacin.

The antibiotic tested in this study was amikacin, which is used in the treatment of many nosocomial infections caused by Gram-negative bacteria, e.g., urinary tract infections or infections associated with the vascular line. In addition, it acts against some Gram-positive bacteria, e.g., methicillin-resistant *Staphylococcus aureus* (MRSA) [18]. Figure 2 shows the structural formula of amikacin.

## 2. Results

Amikacin turned out to be the most active against the strains *Staphylococcus epidermidis* ATCC 12228 and *Staphylococcus aureus* ATCC 25923, the growths of which were inhibited at concentrations of 0.5 and 1 μg/mL, respectively. In turn, a concentration of 2 μg/mL inhibited the growth of the following strains: *Bacillus subtilis* PMC 2021 and *Enterobacter clocae* ATCC 13047, and a concentration of 4 μg/mL was active against the isolates of *Enterococcus faecalis* ATCC 29212 and *Staphylococcus epidermidis* ATCC 35984. An MIC value of 8 μg/mL proved to work against *Escherichia coli* ATCC 25922 and *Proteus mirabilis* ATCC 7002. An amikacin concentration of 16 μg/mL was found to be active against *Pseudomonas aeruginosa* ATCC 27853. The highest concentration inhibiting the growth of microorganisms (64 μg/mL) was recorded for the following strains: *Staphylococcus aureus* ATCC 43300, *Klebsiella pneumoniae* ATCC 27736, and *Acinetobacter baumannii* ATCC 19606 (Table 1). In summary, the median MIC values obtained for amikacin were 3 μg/mL for Gram-positive bacteria and 12 μg/mL for Gram-negative bacteria. The standard deviation was 25.23 and 29 μg/mL for Gram-positive and Gram-negative strains, respectively. The lower and upper quartiles were 1 and 4 μg/mL for Gram-positive strains and 8 and 64 μg/mL for Gram-negative strains. A statistical analysis showed no differences in the obtained MIC values of amikacin (*p* = 0.105) between Gram-positive and Gram-negative bacteria (Table 2).

Rutin hydrate showed antibacterial activity against all the tested strains. The MIC values of RH for Gram-positive strains ranged from 128 to 512 µg/mL with a median of 384 µg/mL, lower quartile of 256 µg/mL, upper quartile of 512 µg/mL, and standard deviation of 170.13 µg/mL. On the other hand, for the Gram-negative bacteria, the median, lower quartile, upper quartile, and standard deviation were 384, 256, 1024, and 400.02 µg/mL, respectively (Table 2). A statistical analysis showed no differences in the obtained MIC values of rutin hydrate (*p* = 0.676) between Gram-positive and Gram-negative bacteria (Table 2). The lowest MIC value of rutin hydrate amounting to 128 μg/mL was recorded for the *Staphylococcus epidermidis* ATCC 35984 and *Enterobacter clocae* ATCC 13047 strains. The highest MIC value (1024 μg/mL) was observed for two strains: *Pseudomonas aeruginosa* ATCC 27853 and *Acinetobacter baumannii* ATCC 19606. An MIC of 256 μg/mL was noted for four strains: *Klebsiella pneumoniae* ATCC 27736, *Proteus mirabilis* ATCC 7002, *Bacillus subtilis* PCM 2021, and *Enterococcus faecalis* ATCC 29212. The MIC was 512 μg/mL for the remaining four isolates: *Staphylococcus aureus* ATCC 25923, *Staphylococcus aureus* ATCC 43300, *Staphylococcus epidermidis* ATCC 12228, and *Escherichia coli* ATCC 25922 (Table 1).

**Figure 3:**
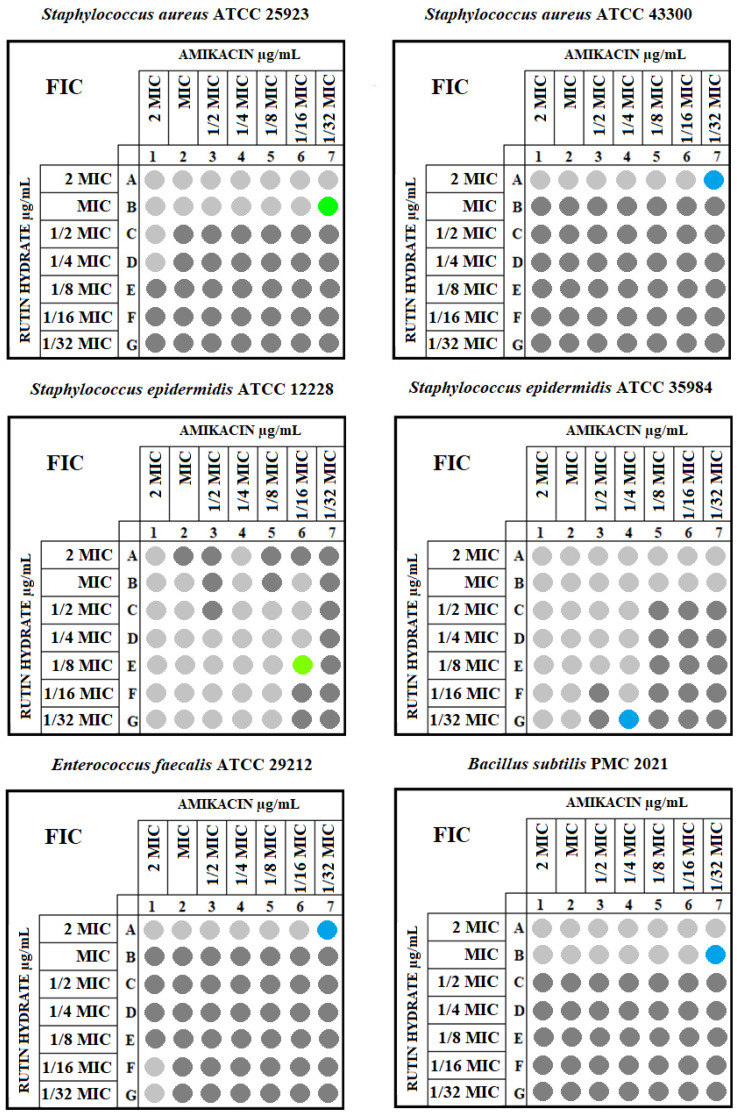

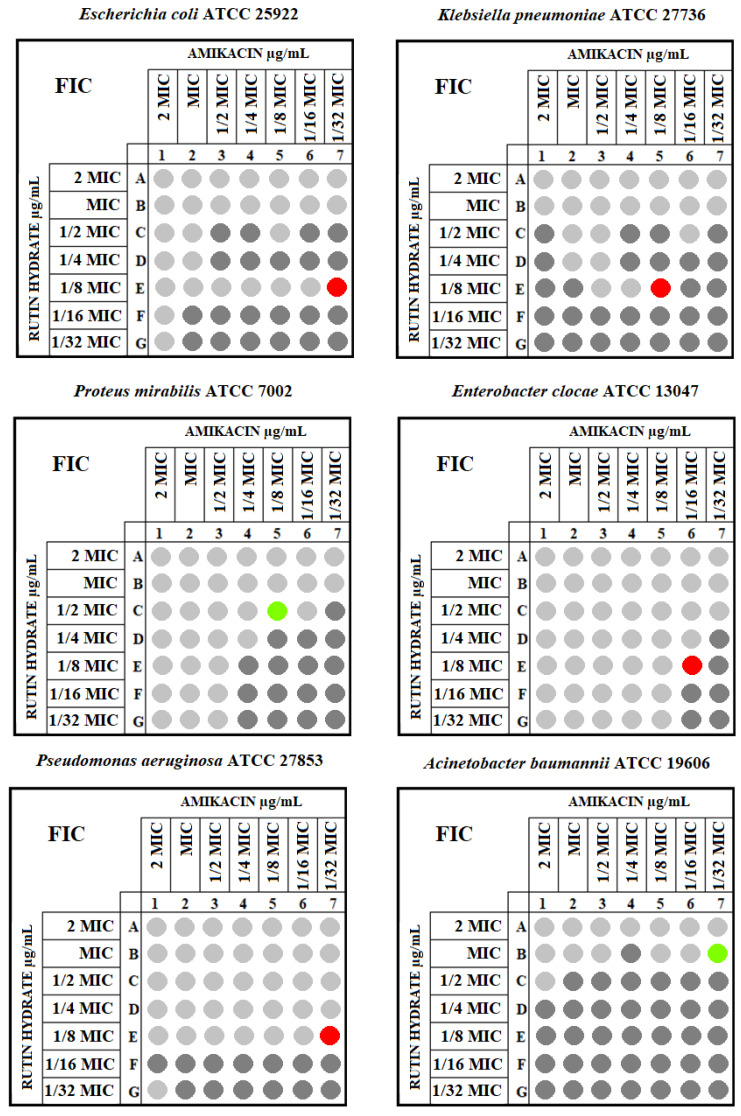
The checkerboard assay for Gram-positive and Gram-negative tested strains. The dark gray shows more than 50% bacterial growth in a well, while the light gray represents less than 50% bacterial growth in a well compared with the growth control. Only light gray wells were used to determine the FIX index. The FIC index for each strain is marked with a color. The blue shows an indifferent, the green an additive, and the red a synergistic interaction. The FIC index was marked on the growth inhibition border and represents the point where a synergistic/additive or indifferent effect was the most visible.

The final stage of the research was the determination of the FIC index values. The results of the checkerboard assay for the reference strains are presented in Figure 3. Moreover, based on the checkerboard, the MIC values were determined for RH in combination with amikacin and for amikacin in combination with RH. The MIC values of amikacin with RH for Gram-positive strains ranged from 0.0625 to 1 µg/mL with a median of 0.19 µg/mL, lower quartile of 0.06 µg/mL, upper quartile 0.62 of µg/mL, and standard deviation of 0.38 µg/mL. For Gram-negative bacteria, the descriptive statistics above were 1.25, 0.5, 2, and 1.46 µg/mL, respectively (Table 2). The amikacin–RH combination turned out to be more active against Gram-negative strains, where in four cases, a synergistic effect was found, and in two, it was additive. In the group of Gram-positive bacteria, two additive interactions were noted, and for the remaining strains, the effect of combining the compounds was indifferent. No antagonistic effect was noted for any of the tested strains. The results of all the above assays are summarized in Table 1. There were no statistically significant differences in the amikacin MIC, and the MIC decreased after the addition of RH between Gram-positive and Gram-negative strains (*p* = 0.226 and *p* = 0.870, respectively). However, to more precisely investigate differences in the action of the “rutin hydrate—amikacin” combination on Gram-positive vs. Gram-negative bacteria, a larger study group should be used. The descriptive and non-parametric statistics for the MICs of amikacin alone and with rutin hydrate for Gram-negative and Gram-positive strains are presented in Table 2.

## 3. Discussion

The need to launch a new antibacterial drug in the era of antibiotic resistance has prompted scientists around the world to return to natural medicine methods and analyze the potential antibacterial mechanisms of action of plant-derived compounds. The antimicrobial potential of polyphenols, especially rutin, has been confirmed in in vitro studies on bacterial cultures [14,15,16,17,19,20,21,22,23,24,25,26,27]. In our study, amikacin, in small concentrations ranging from 0.5 to 16 μg/mL, was active against both Gram-positive and Gram-negative reference strains. Amikacin in its highest concentration (64 μg/mL) inhibited the growth of the following strains: *Staphylococcus aureus* ATCC 43300, *Klebsiella pneumoniae* ATCC 27736, and *Acinetobacter baumannii* ATCC 19606. Due to the resistance of amikacin to most aminoglycoside-modifying enzymes, it is used in the treatment of infections of strains resistant to other aminoglycosides. This antibiotic is the one most frequently applied to semi-synthetic aminoglycoside. However, strains resistant to amikacin have developed over the years. The first acetyltransferases that inactivated amikacin were reported for *P. aeruginosa.* Adenyltransferase, in turn, was found in *K. pneumoniae*, *E. coli*, *Serratia marcescens*, and *Proteus vulgaris*, and phosphotransferases were isolated from *E. coli*. Amikacin is no exception among other antibiotics, and bacteria develop resistance to it as well [28]. The introduction of natural compounds such as rutin hydrate into treatment may increase the potential of amikacin by improving its pharmacokinetic and pharmacodynamic properties, which could lead to a reduction in the doses, and thus reduce side effects associated with its use. This advantage of rutin hydrate as an antibacterial agent may consequently contribute to inhibiting the spread of resistance among bacteria. To the best of our knowledge, studies on the direction of interactions between RH and amikacin have not yet been conducted, but the antibacterial properties of rutin have been studied, and the results of these works are discussed below.

Jhanji et al. in 2021 examined the antimicrobial potential of rutin on four selected reference strains in comparison with commonly used antibiotics, which were streptomycin, ciprofloxacin, and amoxicillin. They observed that the growth of *B. subtilis* in the presence of rutin at a concentration of 256 μg/mL was inhibited at a rate of about 75%. The growth inhibition of *P. aeruginosa* at a concentration of 1024 μg/mL was approximately 90%. In turn, in cultures of *E. coli* and *S. aureus*, a rutin concentration of 512 μg/mL inhibited bacterial growth by approximately 70%. Research conducted by Jhanji et al. showed that the rutin solutions used by them in the concentrations defined in this study as the MIC values inhibited the growth of the indicated bacterial strains in the range of 70–90% [15]. Additionally, Jhanji et al. showed a synergistic effect of the combination “rutin-berberine-streptomycin” against *B. subtilis* (FIC index = 0.25), “rutin-berberine-ciprofloxacin” against *P. aeruginosa* (FIC index = 0.25), and “rutin-berberine-amoxicillin” against *S. aureus* (FIC index = 0.125).

In addition, Jhanji et al., in research conducted in 2020, considered a mechanism of antimicrobial activity in rutin [16]. Researchers have suggested that rutin may inhibit the action of *efflux pumps* and interfere with biofilm formation. Both mechanisms have been confirmed in in silico and in vitro studies on bacterial cultures. Interestingly, in our study, RH turned out to be more active against the reference strain *S. epidermidis* ATCC 35984 (128 μg/mL), which has the ability to form a biofilm, than against the biofilm (-) strain *S. epidermidis* ATCC 12228 (512 μg/mL). Our next studies will focus on assessing the ability to form biofilm under the influence of rutin hydrate.

Wang et al., in their study, focused on the analysis of the antimicrobial potential of rutin against the *K. pneumoniae* ATCC 700603 and *E. coli* ATCC 25922 reference strains. The MIC value of rutin against the *E. coli* strain was 512 μg/mL, which was the same as in this study. In turn, the MIC value for *K. pneumoniae* was 1024 μg/mL, which was four times higher than the concentration of 256 μg/mL obtained in our study against *K. pneumoniae* ATCC 27736. The difference in the obtained MIC values is probably due to the fact that *K. pneumoniae* ATCC 700603, unlike *K. pneumoniae* ATCC 27736, produces extended-spectrum beta-lactamases [17].

In addition, Wang et al., like Jhanji et al., studied the inhibition of biofilm formation in *K. pneumoniae* strains under the influence of rutin. Their research showed that rutin had a strong ability to inhibit biofilm formation in weak, moderate, and strong biofilm-producing strains. The ability to inhibit biofilm formation was confirmed by analyzing the expression profile of 15 genes involved in biofilm formation. A significant reduction in the expression of several genes from this group was observed, which correlated with a reduction in the accumulation of *K. pneumoniae* biomass. A significant inhibition of biofilm formation was observed at rutin concentrations of 512 and 256 μg/mL [17].

The confirmation of the antimicrobial potential of rutin opens many research paths that will be aimed at analyzing the change in the antibacterial properties of rutin in the presence of other polyphenols with a similar effect, as well as antibiotics and various forms of rutin preparation, e.g., in the form of nanoparticles, biomaterials coated with rutin, etc. The validity of searching for compounds against which rutin exhibits synergy and/or potentiates antibacterial activity has been confirmed by research conducted in 2015 by Amin et al. These scientists decided to determine the effect of rutin, morin, and quercetin (in various combinations) with 12 selected antibiotics on 100 clinical MRSA strains and the *S. aureus* ATCC 43300 reference strain. It was found that the tested compounds in combination with antibiotics enhanced the antibacterial effect against the examined staphylococcal strains. The relationship between flavonoids and antibiotics was additive in most cases, but synergism was also observed in a few cases. Moreover, Amin et al. examined potassium release to determine the effect of antibiotic–flavonoid combinations on the cytoplasmic membrane for the tested staphylococci. They proved that flavonoids alone or in combination damage the bacterial cell membrane [24]. The use of a mixture of rutin with other flavonoids may allow for its use in much lower concentrations than the MIC values determined in this study.

The results obtained and presented in this paper confirm the logic and effectiveness of using rutin hydrate as an agent combating antibiotic-resistant bacterial strains. This potential is conditioned by specific mechanisms of action, the study of which is the second step in confirming the usefulness of rutin in bacterial infections. Numerous studies have indicated that rutin, as a polyphenol, modifies the permeability of bacterial cell membranes and changes the stiffness of the cell wall, which leads to the loss of its integrity [25]. In addition, it has been suggested that rutin could alter intracellular functioning by binding hydroxyl residues to important bacterial enzymes [26]. Moreover, it was reported that rutin has the ability to inhibit β-lactamases and biofilm formation [27].

The research results discussed above, as well as the results presented in this paper, indicate the antimicrobial potential of rutin. In addition, they prove that this compound increases the antibacterial potential of antibiotics. Due to the anti-biofilm activity of rutin indicated by several researchers, it seems important to further study this compound in this respect. Moreover, it is necessary to examine the combination “rutin hydrate-amikacin” on clinical strains of Gram-negative bacteria with different resistance mechanisms.

## 4. Materials and Methods

### 4.1. Materials

All reference strains of bacteria came from the ATCC culture bank (American Collection of Cell Cultures, Manassas, VA,, USA), except the strain *Bacillus subtilis* PCM 2021, which was obtained from the Polish Collection of Microorganisms (Wroclaw, Poland). The tests were carried out on twelve selected strains of Gram-positive and Gram-negative bacteria: *Staphylococcus aureus* ATCC 25923, *Staphylococcus aureus* ATCC 43300, *Staphylococcus epidermidis* ATCC 12228, *Staphylococcus epidermidis* ATCC 35984, *Enterococcus faecalis* ATCC 29212, *Bacillus subtilis* PCM 2021, *Escherichia coli* ATCC 25922, *Klebsiella pneumoniae* ATCC 27736, *Proteus mirabilis* ATCC 7002, *Enterobacter cloacae* ATCC 13047, *Pseudomonas aeruginosa* ATCC 27853, and *Acinetobacter baumannii* ATCC 19606. Rutin hydrate was obtained from Sigma Chemical Co. (St. Louis, MO, USA).

### 4.2. Determination of the MIC Values of the Tested Bacterial Strains

In order to determine the lowest bacterial-growth-inhibiting concentrations for rutin hydrate and amikacin, the serial microdilution method was used with sterile, 96-well titer plates (FL Medical, Torreglia, Italy) with a final volume of 200 µL [29,30].

An amount of 100 µL of TSB medium (BTL) was aliquoted into all wells of the titer plates. The next step was to prepare serial dilutions of the tested compounds (from 1024 to 1 µg/mL). Tested compounds were not introduced into the 12th column of the titration plate, which was a growth control. Next, one hundred microliters of mid-logarithmic-phase bacterial cultures (5 × 10^5^ CFU/mL) in TSB was added to each well (rows B–G). Row H wells were used to control the sterility of the medium. In addition, in row A of each plate, a suspension of TSB medium and the tested compound at various concentrations was prepared, which allowed the subsequent measurement of background extinction. Each examined strain was measured in triplicate. The next stage of the study was the incubation of the titer plates at 37 °C for 24 h. After incubation, the optical density of the culture was measured at 595 nm. Absorbance results for individual strains were read using a Multiskan EX reader (Thermo Electron Corp., Vantoa, Finland) [31,32]. MICs were defined as the lowest RH concentrations that completely inhibited bacterial growth [33]. The MIC results obtained in this stage of the research were used to design the “chessboard” and determine the FIC values in the next stage.

### 4.3. Determination of the FIC Values for the Tested Strains

**Figure 4:**
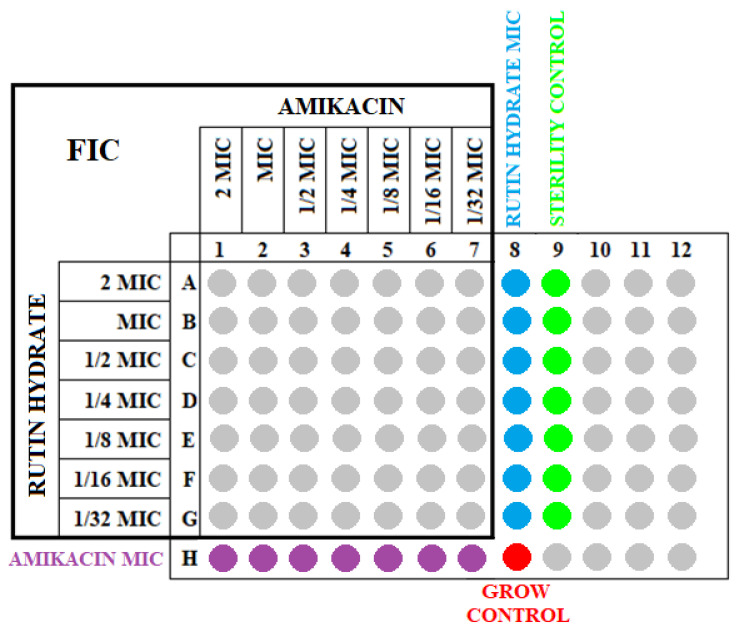
Scheme of the checkerboard method with the simultaneous indication of the FICs and MICs.

The sensitivity of the tested reference isolates to the combination of amikacin with RH was assessed by determining the fractionated inhibitory concentration (FIC) value for each strain. The checkerboard microdilution method with modifications [34,35] was used to determine the total susceptibility effect of the tested strains. First, the solutions of the antibiotic and RH were prepared, corresponding to 8 MIC values (according to the MIC values obtained in the first stage of the study). A series of 1/8 MIC dilutions were then produced. An amount of 95 µL of double-concentrated Mueller–Hinton (BLT) medium was added to each well of the titration plate. Then, 50 µL of RH and the appropriate concentration of amikacin were pipetted. Finally, 5 µL of a 0.5 McFarland bacterial suspension was added. The final volume of each well was 200 µL. Therefore, the solutions corresponding to the concentrations of 8 MICs, 4 MICs, 2 MICs, 1 MIC, 1/2 MIC, 1/4 MIC, and 1/8 MIC were prepared for amikacin and RH to account for the dilution of 50 µL of these substances in 200 µL of the solution and were obtained as follows: 2 MICs, 1 MIC, 1/2 MIC, 1/4 MIC, 1/8 MIC, 1/16 MIC, and 1/32 MIC in each of the wells. At the last column and row, respectively, instead of RH and amikacin, 50 µL of medium was added. The scheme of the “chessboard” plate is shown in Figure 4.

The plates prepared in this way were incubated in an aerobic incubator at 37 °C for 24 h. The absorbances at 595 nm were then read. Next, the percentage increase in individual wells relative to the growth control was calculated. Only wells that represented less than 50% bacterial growth compared with the growth control were considered for FIC index determination.

The MICs for rutin hydrate and amikacin for each strain were then recalculated. At the end, the FICs (fractional inhibitory concentrations) were calculated, i.e., the fractional inhibitory concentration for RH and amikacin and their sum for each well. The following formula was used:

FIC index = FICA + FICB = (MICA + B)/ MICA + (MICB + A)/MICB,

where:

MICA + B—MIC of the antibiotic in the presence of a polyphenolic compound;
MICB + A—MIC of a polyphenolic compound in the presence of an antibiotic;
MICA—MIC of the antibiotic itself;
MICB—MIC of the polyphenolic compound itself.

Next, on the growth inhibition border, the FIC index was established for each strain. The FIC index was the point where synergistic/additive or indifferent effects were the most visible.

Based on the FIC index value for each strain, the relationship between rutin hydrate and amikacin was assessed according to the following scale:

FIC ≤ 0.5—synergism;
0.5 < FIC ≤ 1—additive action;
1 < FIC ≤ 4—indifference;
FIC > 4—antagonism [35].

### 4.4. Statistical Analysis

In order to check whether there were differences in the obtained MICs of RH or amikacin values between Gram-positive and Gram-negative bacteria, the Mann–Whitney U test was used for two independent samples. The same test determined whether there were statistically significant differences in the amikacin MICs, and the MICs decreased after the addition of RH between Gram-positive and Gram-negative bacterial strains. Statistical calculations were made in the Statistica 13.0 program. The level of statistical significance was *p* < 0.05.

## 5. Conclusions

The results of the presented study demonstrate that rutin hydrate possesses antibacterial properties against the tested reference strains, and this effect is similar for both Gram-positive and Gram-negative bacteria. The combination of “amikacin–RH” was effective against Gram-negative bacteria, where in four cases, a synergistic effect was found, and in two, it was additive. On the other hand, two additive interactions and four indifferent effects were noted for Gram-positive bacteria. The in vitro synergistic interaction of RH and amikacin suggests that this effect could also be observed in vivo. Future studies should focus on the mechanism of action of RH on bacterial cells.

## Figures and Tables

**Table 1:** The MIC values of RH, amikacin alone, and amikacin in combination with RH towards examined strains, along with the obtained FIC index values and their interpretations.

Strain	Gram	MIC of Rutin Hydrate [μg/mL]	MIC of Amikacin [μg/mL]	MIC of Amikacin with Rutin Hydrate [μg/mL]	FIC Index	Interaction
*Staphylococcus aureus* ATCC 25923	Positive	512	1	0.0625	0.563	additive
*Staphylococcus aureus* ATCC 43300	Positive	512	64	1	1.016	indifference
*Staphylococcus epidermidis* ATCC 12228	Positive	512	0.5	0.25	0.656	additive
*Staphylococcus epidermidis* ATCC 35984	Positive	128	4	4	1.013	indifference
*Enterococcus faecalis* ATCC 29212	Positive	256	4	0.125	1.031	indifference
*Bacillus subtilis* PMC 2021	Positive	256	2	0.0625	2.031	indifference
*Escherichia coli* ATCC 25922	Negative	512	8	0.125	0.078	synergism
*Klebsiella pneumoniae* ATCC 27736	Negative	256	64	4	0.188	synergism
*Proteus mirabilis* ATCC 7002	Negative	256	8	2	0.75	additive
*Enterobacter clocae* ATCC 13047	Negative	128	2	0.5	0.5	synergism
*Pseudomonas aeruginosa* ATCC 27853	Negative	1024	16	0.5	0.094	synergism
*Acinetobacter baumannii* ATCC 19606	Negative	1024	64	2	0.532	additive

**Table 2:** Descriptive and non-parametric statistics for the MIC values of rutin hydrate, amikacin alone, and amikacin with the addition of rutin hydrate for Gram-negative and Gram-positive strains.

	MIC of Rutin Hydrate [μg/mL]		MIC of Amikacin [μg/mL]		MIC of Amikacin with Rutin Hydrate [μg/mL]	
	Gram-Positive Strains	Gram-Negative Strains	Gram-Positive Strains	Gram-Negative Strains	Gram-Positive Strains	Gram-Negative Strains
Median	384	384	3	12	0.19	1.25
Standard deviation	170.13	400.02	25.23	29	0.38	1.46
Lower quartile	256	256	1	8	0.06	0.5
Upper quartile	512	1024	4	64	0.62	2
Mann–Whitney U test	*p* = 0.676		*p* = 0.105		*p* = 0.226	

## Data Availability

Not applicable.
